# Hematopoietic cell transplantation and cellular therapies in Europe 2022. CAR-T activity continues to grow; transplant activity has slowed: a report from the EBMT

**DOI:** 10.1038/s41409-024-02248-9

**Published:** 2024-03-04

**Authors:** Jakob R. Passweg, Helen Baldomero, Fabio Ciceri, Rafael de la Cámara, Bertram Glass, Raffaella Greco, Mette D. Hazenberg, Krzysztof Kalwak, Donal P. McLornan, Bénédicte Neven, Zinaida Perić, Antonio M. Risitano, Annalisa Ruggeri, John A. Snowden, Anna Sureda

**Affiliations:** 1 EBMT Activity Survey Office, Hematology Division, University Hospital, Basel, Switzerland; 2 Unit of Hematology and Bone Marrow Transplantation, IRCCS San Raffaele Hospital, Vita-Salute San Raffaele University, Milan, Italy; 3 Hematology Department, Hospital Universitario de la Princesa, Madrid, Spain; 4 Klinik für Hämatologie und Stammzelltransplantation, HELIOS Klinikum Berlin-Buch, Berlin, Germany; 5 Department of Hematology, Amsterdam University Medical Centres, University of Amsterdam, Amsterdam, The Netherlands; 6 Clinical Department of Pediatric BMT, Hematology and Oncology, Wroclaw Medical University, Wroclaw, Poland; 7 Department of Haematology, University College London Hospitals NHS Foundation Trust, London, UK; 8 Pediatric immune-hematology unit, Necker Children Hospital, Assistance Publique Hôpitaux de Paris, Paris, France; 9 School of Medicine, University of Zagreb, University Hospital Center Zagreb, Zagreb, Croatia; 10 Hematology and Hematopoietic Transplant Unit, Azienda Ospedaliera di Rilievo Nazionale “San Giuseppe Moscati” (A.O.R.N. Giuseppe Moscati), Avellino, Italy; 11 Department of Haematology, Sheffield Teaching Hospitals NHS Foundation Trust, Sheffield, UK; 12 Clinical Hematology Department, Institut Català d’Oncologia-Hospitalet, Institut d’Investigació Biomèdica de Bellvitge (IDIBELL), University of Barcelona, Barcelona, Spain

**Keywords:** Haematological diseases, Haematological cancer

## Abstract

In 2022, 46,143 HCT (19,011 (41.2%) allogeneic and 27,132 (58.8%) autologous) in 41,854 patients were reported by 689 European centers. 4329 patients received advanced cellular therapies, 3205 of which were CAR-T. An additional 2854 patients received DLI. Changes compared to the previous year were an increase in CAR-T treatments (+27%) and decrease in allogeneic (−4.0%) and autologous HCT (−1.7%). Main indications for allogeneic HCT were myeloid malignancies (10,433; 58.4%), lymphoid malignancies (4,674; 26.2%) and non-malignant disorders (2572; 14.4%). Main indications for autologous HCT were lymphomas (7897; 32.9%), PCD (13,694; 57.1%) and solid tumors (1593; 6.6%). In allogeneic HCT, use of sibling donors decreased by −7.7%, haploidentical donors by −6.3% and unrelated donors by −0.9%. Overall cord blood HCT decreased by −16.0%. Use of allogeneic, and to a lesser degree autologous HCT, decreased for lymphoid malignancies likely reflecting availability of new treatment modalities, including small molecules, bispecific antibodies, and CAR-T cells. Pediatric HCT activity remains stable (+0.3%) with differences between allogeneic and autologous HCT. Use of CAR-T continues to increase and reached a cumulative total of 9039 patients treated with wide differences across European countries. After many years of continuous growth, increase in application of HCT seems to have slowed down.

## Introduction

The European Society for Blood and Marrow Transplantation (EBMT) published a survey in 1990 [1] describing activity in hematopoietic cell transplantation (HCT) centers in Europe, updated annually thereafter. The survey, now spanning 33 years, includes patients receiving more than 940,000 transplants. The survey was designed in the form of a single page spreadsheet for ease of reporting and has remained in this format ever since. Many additional features have been added, such as refined disease classification, donor type and stem cell source, information on conditioning intensity and separating pediatric activity.

HCT is an established procedure for many acquired or inherited disorders of the hematopoietic system, benign or neoplastic, including those of the immune system, and to facilitate enzyme replacement in metabolic disorders [2–4]. The activity survey of the EBMT, describing the status of HCT, has become an instrument to observe trends and monitor changes in HCT technology in Europe and neighboring countries [5–15]. The survey, using a standardized structure, captures the numbers of HCT from highly committed participating centers, stratified by indication, donor type and stem cell source over time (https://www.ema.europa.eu/en/documents/scientific-guideline/qualification-opinion-cellular-therapy-module-european-society-blood-marrow-transplantation-ebmt_en.pdf) [16–18]. In more recent years, the survey also included information on cellular therapies qualifying as medicinal products with hematopoietic cells for uses other than to replace the hematopoietic system [19]. The analysis of the survey data since 1990 has illustrated a continued and constant increase in the annual numbers of HCT and transplant rates for both allogeneic and autologous HCT. A drop in activity was noted in 2020 for the first time, likely driven by the SARS-CoV-2 pandemic [14]. This 2022 survey data show that after some recovery in the number of patients treated in the second year of the pandemic, the previous trend of continuous growth appears to have slowed down.

## Patients and methods

### Data collection and validation

We invited participating centers to report their data for 2022 using the activity survey as shown in Table 1. Patients receiving their first transplant in the survey year are reported by disease, donor type and stem cell source. Additional information on the numbers of subsequent transplants performed due to relapse, rejection, or those that are part of a planned sequential protocol are reported in summative form. Information on the number of patients receiving un-manipulated donor lymphocyte infusions (DLIs), non-myeloablative or reduced intensity HCT, and the number of pediatric HCT were also collected.

**Table 1:** Numbers of patients receiving a first allogeneic or first autologous HCT in 2022 by indication, donor type and stem cell source.

	TRANSPLANT ACTIVITY 2022																	
	No. of patients																	
	Allogeneic												Autologous			Total		
	Family									Unrelated						Allo	Auto	Total
	HLA-id			Twin	Haplo > = 2MM		Other family						BM	BM + PBPC	Cord			
	BM	PBPC	Cord	all	BM	PBPC	BM	PBPC	Cord	BM	PBPC	Cord						
**Myeloid malignancies**	**226**	**2160**	**3**	**4**	**227**	**1517**	**6**	**65**	**0**	**232**	**5873**	**120**	**2**	**206**	**0**	**10433**	**208**	**10641**
Acute myeloid leukemia	166	1515	1	4	181	1100	4	50	0	135	3713	92	1	205	0	6961	206	7167
1st complete remission	117	1008	0	2	113	626	2	37	0	95	2120	61	1	168	0	4181	169	4350
not 1st complete remission	34	346	1	2	48	305	2	10	0	26	842	24	0	33	0	1640	33	1673
AML therapy related or myelodysplasia related changes	15	161	0	0	20	169	0	3	0	14	751	7	0	4	0	1140	4	1144
Chronic myeloid leukemia	13	82	1	0	5	38	0	1	0	4	183	4	0	0	0	331	0	331
chronic phase	7	42	0	0	2	16	0	1	0	1	93	2	0	0	0	164	0	164
not chronic phase	6	40	1	0	3	22	0	0	0	3	90	2	0	0	0	167	0	167
MDS or MD/MPN overlap	44	379	1	0	29	284	2	10	0	82	1427	23	1	1	0	2281	2	2283
MPN	3	184	0	0	12	95	0	4	0	11	550	1	0	0	0	860	0	860
**Lymphoid malignancies**	**253**	**1116**	**5**	**5**	**206**	**806**	**5**	**36**	**1**	**210**	**1982**	**49**	**12**	**21626**	**0**	**4674**	**21638**	**26312**
Acute lymphatic leukemia	224	700	5	3	161	497	5	26	1	188	1206	35	0	40	0	3051	40	3091
1st complete remission	128	503	3	1	65	262	4	10	0	84	807	17	0	39	0	1884	39	1923
not 1st complete remission	96	197	2	2	96	235	1	16	1	104	399	18	0	1	0	1167	1	1168
Chronic lymphocytic leukemia	1	34	0	0	2	30	0	0	0	4	85	1	0	7	0	157	7	164
Plasma cell disorders - MM	1	55	0	1	5	24	0	1	0	1	73	0	1	13269	0	161	13270	13431
Plasma cell disorders - other	1	6	0	0	0	1	0	0	0	1	17	0	1	423	0	26	424	450
Hodgkin lymphoma	8	92	0	0	18	99	0	2	0	5	121	3	8	2210	0	348	2218	2566
DLBCL NHL all types	3	76	0	0	6	52	0	2	0	1	124	0	0	2841	0	264	2841	3105
Other B-cell NHL	2	43	0	0	2	31	0	2	0	2	111	2	2	2029	0	195	2031	2226
T-cell NHL	13	110	0	1	12	72	0	3	0	8	245	8	0	807	0	472	807	1279
**Solid tumors**	**1**	**1**	**0**	**1**	**1**	**21**	**0**	**0**	**0**	**1**	**2**	**0**	**16**	**1576**	**1**	**28**	**1593**	**1621**
Neuroblastoma	1	0	0	1	1	21	0	0	0	0	1	0	13	533	0	25	546	571
Soft tissue sarcoma/Ewing sarcoma	0	1	0	0	0	0	0	0	0	0	0	0	1	231	0	1	232	233
Germinal tumors	0	0	0	0	0	0	0	0	0	0	1	0	0	428	0	1	428	429
Other solid tumors	0	0	0	0	0	0	0	0	0	1	0	0	2	384	1	1	387	388
**Non malignant disorders**	**745**	**363**	**13**	**4**	**144**	**178**	**65**	**54**	**1**	**463**	**496**	**46**	**1**	**475**	**2**	**2572**	**478**	**3050**
Bone marrow failure - SAA	180	141	0	3	46	52	10	6	0	171	170	8	0	0	0	787	0	787
Bone marrow failure - other	66	35	2	0	13	19	8	10	1	71	48	5	0	0	0	278	0	278
Thalassemia	174	40	3	0	4	9	10	10	0	32	73	1	1	7	0	356	8	364
Sickle cell disease	144	93	8	1	35	18	12	3	0	11	8	0	0	2	0	333	2	335
Primary Immune deficiencies	147	45	0	0	35	74	23	19	0	137	145	11	0	2	2	636	4	640
Inherited disorders of metabolism	20	7	0	0	10	6	2	5	0	37	45	21	0	0	0	153	0	153
Auto immune disease - MS	0	1	0	0	0	0	0	0	0	0	0	0	0	380	0	1	380	381
Auto immune disease -SSC	0	0	0	0	0	0	0	0	0	0	0	0	0	61	0	0	61	61
Auto immune disease -other	14	1	0	0	1	0	0	1	0	4	7	0	0	23	0	28	23	51
Others	29	16	0	0	13	15	4	6	0	21	46	5	0	75	0	155	75	230
**TOTAL PATIENTS**	**1254**	**3656**	**21**	**14**	**591**	**2537**	**80**	**161**	**2**	**927**	**8399**	**220**	**31**	**23958**	**3**	**17862**	**23992**	**41854**
Re/additional transplants	23	135	0	2	45	307	5	15	0	50	537	30	2	3138	0	1149	3140	4289
**TOTAL TRANSPLANTS**	**1277**	**3791**	**21**	**16**	**636**	**2844**	**85**	**176**	**2**	**977**	**8936**	**250**	**33**	**27096**	**3**	**19011**	**27132**	**46143**
Patients <18 at HCT	991	396	17	4	312	506	81	72	1	699	913	138	23	1297	2	4130	1322	5452

The bold text indicates the subtotals of the data listed below in standard text until the next subtotal.

In addition, in Table 2, centers reported information on different types of cellular therapies qualifying as advanced therapy medicinal products (ATMP). These therapies result from substantial manipulations of collected cells, whether manufactured by industry centrally or locally by an academic institution.

**Table 2:** Numbers of patients treated with non HCT cellular therapies in 2022 by indication, donor type and cell source.

Number of patients	DLI	CART		MSC		NK cells		Selected/expanded T cells or CIK		Regulatory T cells (TREGS)		Genetically modified T cells		Dendritic cells		Expanded CD34+ cells		Genetically modified CD34+ cells		Other		*Total excluding DLI*	
		Allo	Auto	Allo	Auto	Allo	Auto	Allo	Auto	Allo	Auto	Allo	Auto	Allo	Auto	Allo	Auto	Allo	Auto	Allo	Auto	*allo*	*auto*
GvHD				273	1			1		30										13		317	1
Graft enhancement				30				16								12			2	111	50	169	52
Autoimmune dis.			9	11	3											5				4		20	12
Genetic disease																	4	3	19	3		6	23
Infection				1	8			116	46	4		7	2							6	1	134	57
Malignancy - ALL		44	336			19		18	9				4		4	1				14		96	353
Malignancy - HL/NHL		1	2258			4	2	23				1		1						1	8	31	2268
Malignancy - Myeloma		3	467			7							6							3	12		485
Any other indication		4	83	15	13	15	3	41	17				19		20				4	35	23	110	182
DLI for graft enhancement/failure	804																						
DLI for residual disease	393																						
DLI for relapse	1294																						
DLI per protocol	363																						
**Total**	**2854**	**52**	**3153**	**330**	**25**	**45**	**5**	**215**	**72**	**34**	**0**	**8**	**31**	**1**	**24**	**18**	**4**	**3**	**25**	**190**	**94**	**896**	**3433**

The bold text indicates the subtotals of the data listed below in standard text until the next subtotal.

Quality control measures included several independent systems: confirmation of validity of data entered by the center, selective comparison of the survey data with MED-A data sets in the EBMT Registry database and crosschecking with National Registries.

### Participating centers

Since 1990, a directory of HCT centers consisting of both members of the EBMT and non-members, in both European and collaborating non-European countries has been accrued. The directory is updated annually according to the center’s current activity. In 2022, 731 centers from 54 countries were contacted (44 European and 10 collaborating countries); of which 689 centers responded. This corresponded to a 94.4% return rate and included 15.5% EBMT non-members. Forty-two active centers failed to report in 2022. Participating centers are listed in the supplementary online appendix in alphabetical order, by country, city, and EBMT center code, with their reported numbers of first and total HCT, and of first allogeneic and autologous HCT. The WHO regional office definitions were used to classify countries as European or non-European. Nine collaborating non-European countries participated in the 2022 survey: Algeria, Iran, Iraq, Lebanon, Nigeria, Saudi Arabia, South Africa, Tunisia, and United Arab Emirates. Their data, 2714 HCT in 2601 patients, from 28 actively transplanting centers made up 5.9% of the total data set and are included in all analyses.

### Patient and transplant numbers

Wherever appropriate, patient numbers corresponding to the number of patients receiving a first transplant in 2022, and transplant numbers reflecting the total number of transplants performed were listed. The term sibling donor included HLA identical siblings and twins but not siblings with HLA mismatches. Haploidentical transplants were described as any family member with a full haplotype mismatch. Other family member donors were those related donors that are mismatched to a lesser degree than a full haplotype. For the purpose of the analysis, we added the small number of “other family donors” to haploidentical donor HCT. Unrelated donor transplants included HCT from matched or mismatched unrelated donors. Stem cell source included cells collected from bone marrow, peripheral blood or cord blood. Additional non-first transplants included multiple transplants defined as subsequent transplants within a planned double or triple autologous or allogeneic transplant protocol, and re-transplants (autologous or allogeneic) defined as unplanned HCT for either rejection, poor-graft function, or relapse after a previous HCT.

### Hematopoietic advanced cellular therapies other than hematopoietic cell transplantation

Centers reported patients receiving cellular therapies other than HCT. Hematopoietic advanced cellular therapies were defined as infusion of cells undergoing substantial manipulation after collection, either selection and/or expansion, or genetic modification and thus qualify as investigational or ATMPs. according to Regulation (EC) N° 1394/2007. In this context, “substantial” should be understood as referring to the definition included in the Regulation and subsequent regulatory documents and may not reflect the workload assumed by cell processing facilities working in conjunction with clinical programs. Depending on their nature and indications, hematopoietic cellular therapies may be designed to replace or to complement HCT. Administration of non-substantially manipulated hematopoietic cells, such as transplantation of CD34+ selected hematopoietic stem cells were counted as HCT and not as cellular therapy [18]. Similarly, un-manipulated lymphocyte infusions post-HCT were counted as DLI and not as ATMPs. Hematopoietic cellular therapies include immune effector cells as defined in FACT-JACIE standards for Hematopoietic Cellular Therapy: “A cell that has differentiated into a form capable of modulating or effecting a specific immune response.” This definition covers chimeric antigen receptor T-cells (CAR-T) cells and forms the basis for accreditation requirements in recent EBMT-JACIE recommendations (https://www.ema.europa.eu/en/documents/scientific-guideline/qualification-opinion-cellular-therapy-module-european-society-blood-marrow-transplantation-ebmt_en.pdf) [17].

Hematopoietic cellular therapies were categorized as CAR -T, in vitro selected/ and or expanded T-cells or cytokine activated, such as virus specific T-cells; cytokine-induced killer cells (CIK); regulatory T-cells (TREGS); genetically modified T-cells other than CAR-T; natural killer cells (NK); dendritic cells; mesenchymal stromal cells; in vitro expanded CD34+ cells; and genetically modified CD34+ cells. This survey did not include cells from sources other than hematopoietic tissue. On the other hand, gene therapy protocols, such as those used to treat thalassemia or SCID were included, however reported numbers have remained low to date.

### Transplant and cellular therapy rates

Transplant rates, defined as the total number of HCT per 10 million inhabitants, were computed for each country, without adjusting for patients receiving their HCT in a foreign country. Cellular therapy rates were defined as the numbers of patients receiving a CAR-T cellular therapy treatment per 10 million inhabitants. Population numbers for the European countries in 2022 were obtained from Eurostats: (https://ec.europa.eu/eurostat) and the World Bank database for the non-European countries: (https://databank.worldbank.org).

### Analysis

Wherever appropriate, the absolute numbers of transplanted patients, number of transplants or transplant rates are shown for specific countries, indications, or transplant techniques. Myeloid malignancy includes acute myeloid leukemia (AML), myelodysplastic or myelodysplastic/myeloproliferative neoplasia (MDS or MD/MPN overlap), myeloproliferative neoplasm (MPN), and chronic myeloid leukemia (CML). Lymphoid malignancy includes acute lymphocytic leukemia (ALL), chronic lymphocytic leukemia (CLL), Hodgkin lymphoma (HL), non-Hodgkin lymphoma (NHL) and plasma cell disorders (PCD) (including multiple myeloma (MM) and other PCD). Non-malignant disorders include bone marrow failure (BMF: severe aplastic anemia (SAA) and other BMF), thalassemia and sickle cell disease (HG), primary immune deficiencies (PID), inherited diseases of metabolism (IDM), and autoimmune diseases (AID). Others include histiocytosis and other rare disorders.

## Results

### Participating centers in 2022

Of the 689 centers, 452 (65.6%) performed both allogeneic and autologous transplants; 222 (32.2%) restricted their activity to autologous HCT, and 11 (1.6%) to allogeneic transplants only. Four (0.6%) of the 689 responding centers reported no activity due to renovation or changes within the transplant unit. Within the 685 actively transplanting centers in 2022, 440 (64.2%) performed transplants on adults only and 121 (17.7%) performed transplants on both adult and pediatric patients. An additional 124 (18.1%) were performed in dedicated pediatric transplant centers. Forty-two centers failed to report in 2022 for various reasons, mostly staff shortage. When compared with previously reported data from these centers, it accounts for approximately 1650 non-reported HCTs.

### Numbers of patients, transplants, and trends in 2022

**Fig. 1:**
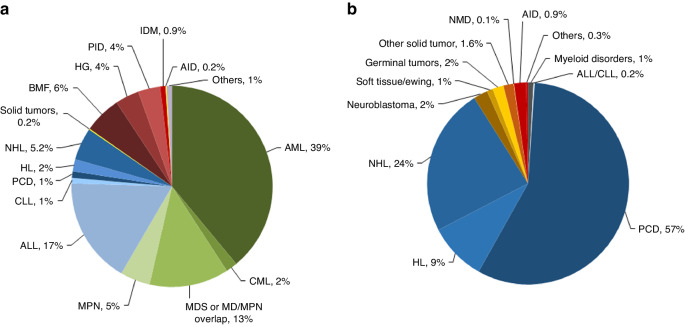
Relative proportion of disease indications for HCT in 2022. Green shades: myeloid malignancies, blue: lymphoid malignancies, brown: solid tumors and red: non malignant disorders. **a** Allogeneic 1st HCT. **b** Autologous 1st HCT.

**Fig. 2:**
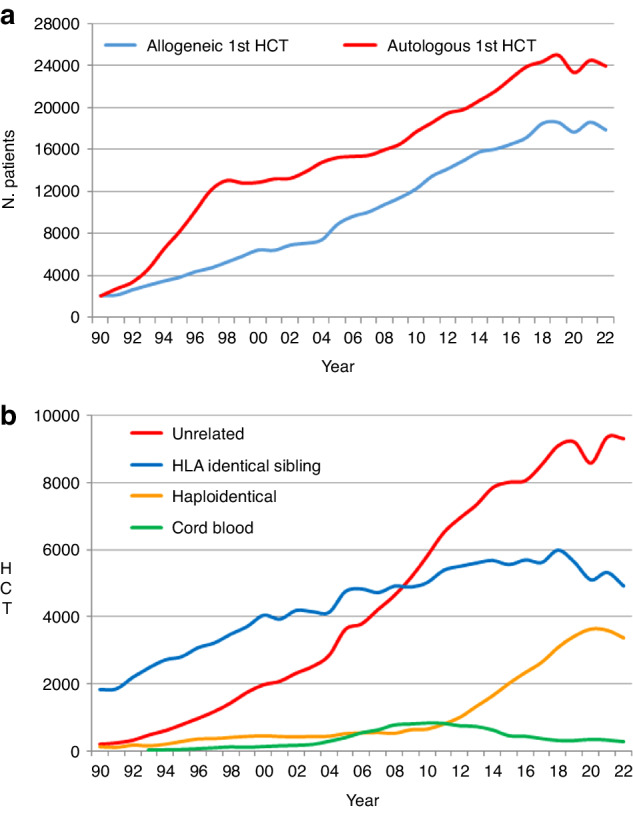
Absolute numbers of patients treated in the years 1990 to 2022. **a** Absolute numbers of patients receiving a first allogeneic or autologous HCT from 1990 to 2022. **b** Change in choice of donor from 1990 to 2022 (1st HCT).

In 2022, 46,143 transplants were reported in 41,854 patients; of these, 19,011 HCT (41.2%) were allogeneic and 27,132 (58.8%) autologous (Table 1 and Fig. 1a, b). After the decrease in HCT activity due to the SARS-CoV-2 pandemic reported in the 2020 survey (14), the total number of transplants increased again in 2021 by +4.5% (+5.4% allogeneic HCT and +3.9% autologous HCT) to 47,412 (15). However, in 2022 the pre-pandemic trend of increasing transplant numbers slowed down, and an overall decrease of −2.7% (−4.0% allogeneic and −1.7% autologous HCT) was seen when compared to 2021. (Figure 2a).

In addition, there were 4289 second or subsequent transplants, 1149 being allogeneic, mainly to treat relapse or graft failure and 3140 autologous, the majority of which were part of multiple transplant procedures such as tandem procedures, to treat relapse, or as salvage autologous transplants for PCD. Furthermore, 573 of the allogeneic HCTs were reported as being given after a previous autologous HCT and were mainly for lymphoma (*n* = 331) or PCD (*n* = 123).

### Pediatric transplantation

The number of pediatric patients (<18 years old at transplant) transplanted in both dedicated pediatric and joint adult-pediatric units was 5452 (4130 allogeneic (76%) and 1322 autologous) (Table 1). This is an overall increase of +0.3% in the total number of transplants, with an increase of +2.5% in allogeneic HCT but a decrease of −6.2% in autologous HCT compared to 2021. Within allogeneic HCT, bone marrow stem cells were used in 2083 patients (50.4%), of which 33.6% were from unrelated donors and peripheral blood stem cells in 1891 patients (45.8%), of which 48.3% were from unrelated donors. Cord blood stem cells were used in 156 pediatric patients (3.8%) which accounts for 57.1% of the total cord blood transplant activity in 2022 (*n* = 273). Of the 156 pediatric cord HCT’s, 138 HCT (88.5%) were from unrelated donors. Due to the design of the survey, detailed analysis by diagnosis is limited to the dedicated pediatric centers only. Here 124 pediatric centers in 27 countries reported 3842 patients, 2967 allogeneic (77.2%) and 875 autologous (22.8%). Main indications for allogeneic HCT were AML (*n* = 422; 70% in early stage), ALL (*n* = 827; 45.9% in early stage) and non-malignant disorders (NMD) (*n* = 1464; 49%), of which 463 (31.6%) were for primary immune deficiencies. There were 1622 family and 1345 unrelated donor HCTs reported. Within the family donors, 660 (40.7%) were from a haploidentical relative. Bone marrow was used as the stem cell source in 1498 patients receiving an allogeneic HCT, of which 998 (66.6%) were family donors. Peripheral blood stem cells were used in 1363 patients with a slightly higher proportion seen in unrelated donors (*n* = 755; 55.4%) when compared to family donors (*n* = 608), and 106 were performed with cord blood. The main indications for autologous HCT were solid tumors, with 749 HCT reported in 2022, primarily for neuroblastoma (*n* = 379, 50.6%). Peripheral blood stem cells were used in the majority of autologous HCT (*n* = 855; 97.7%) with 17 patients receiving bone marrow stem cells and 3 cord blood.

### Main indications

Indications for HCT in 2022 are listed in detail in Table 1. Main indications for allogeneic HCT were myeloid malignancies; 10,433 (AML, CML, MDS or MD/MPN overlap and MPN). For autologous HCT, the main indications were lymphoid malignancies; 21,638 (ALL, CLL, PCD, HL and NHL). Figure 1 shows the distribution of disease indication for allogeneic HCT (Fig. 1a) and autologous HCT (Fig. 1b).

### Changes in allogeneic HCT 2021 to 2022

**Fig. 3:**
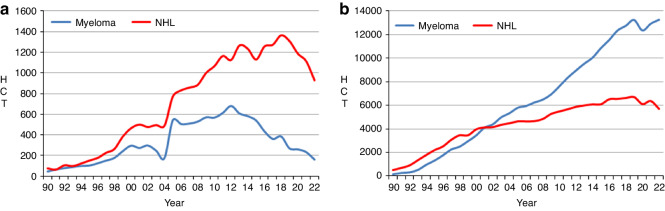
Absolute numbers of HCT for Myeloma and NHL from 1990 to 2022. **a** allogeneic 1st HCT. **b** autologous 1st HCT.

In the report on the 2020 transplant activity during the first year of the SARS-CoV-2 pandemic, decreases were seen for the majority of disease indications when compared to 2019. In 2021, despite the ongoing pandemic in many regions in and around Europe, an increase in transplant activity for many indications, where decreases were previously reported, was observed. However, this year’s 2022 activity report shows again a decrease for several indications where increases were reported previously. When compared to the 2021 activity, an overall decrease in all allogeneic HCT of −4.0% is observed vs the increase of +5.4% reported in 2021 (Fig. 2a). AML, the leading indication for allogeneic HCT, accounting for 39% of all allogeneic HCT, decreased by −2.3% (+3.9% in 2021). A decrease of −2.0% is seen in early stage disease (+6.3% in 2021) while allogeneic HCT for therapy-related AML or AML with myelodysplasia-related changes continued to increase by +5.4% (+6.5% in 2021). Allogeneic HCT for advanced stage AML however, continued to decrease by −7.6% (−2.9% in 2021). After the increase of +12.4% observed in 2021 for allogeneic HCT for CML, a decrease of −16.8% was seen, showing a continuation of the overall downward trend of −84.5% seen since the peak of activity reported in 2000. Allogeneic HCT for MDS or MD/MPN overlap decreased by −5.5% (+9.2% in 2021) while MPN continued to increase by +6.0% (+1.9% in 2021). HCT for ALL, comprising 17% of all allogeneic HCT, decreased overall by −3.4%, (+4.0% in 2021), and primarily in early stage with −5.4% (+7.7% in 2021). In both CLL and HL, transplants continued to decrease by −16.9% and −15.1%, respectively, in contrast to the increase reported in 2021 of +11.8% and +9.3% respectively. In addition, a decrease of −30.9% was observed again in allogeneic HCT activity for myeloma (−10.0% in 2021). Within NHL a continued decrease of −16.5% was observed (−6.2% in 2021) which has been decreasing continuously since 2019 potentially indicating a trend towards utilization of other available treatments (Fig. 3a). Allogeneic transplant numbers for NHL were 264 for DLBCL (all types), 195 for all other B-cell lymphomas and 472 for T-cell NHL. Within the non-malignant disorders, an overall increase of +2.8% was reported (+13% in 2021). BMF-SAA activity increased by +9.3% (+6.5% in 2021) and BMF-non-SAA by +4.5% (+17.7% in 2021). Within the hemoglobinopathies, the number of allogeneic HCT for thalassemia increased again by +23.2% (+5.1% in 2021) and for sickle cell disease by +2.8% (+44.6% in 2021). Reported activities for PID and IDM decreased in 2022, PID by −5.5% (+8.4% in 2021) and IDM by −26.8% (+20.8% in 2021). Of all allogeneic HCT, 7772 (41%) were performed using non-myeloablative or reduced intensity conditioning in 2022.

### Changes in donor type and stem cell source 2021 to 2022

In 2022, an overall decrease was reported in all types of donor choice (Fig. 2b). The overall numbers of patients treated with family donors decreased by −7.1% (+2.0% in 2021). HLA identical sibling and syngeneic twin donors decreased by −7.7% (+4.2% in 2021) and haploidentical donors by −6.3% (−1.2% in 2021), 80% of which are from peripheral blood and 20% from marrow as a stem cell source. For unrelated donors a small decrease of −0.9% compared to the increase of +8.7% reported in 2021 was seen. In the 2020 survey, we observed for the first time in several years that the rate of cord blood HCT for all donor types increased by +11.7% from 309 to 345 and mainly included unrelated cord blood HCT (86%). However, in 2021, the decrease of −5.8% (325 cord blood HCT) continued into 2022 with a further decrease of −16.0% to 273 cord blood HCT.

### Changes in autologous HCT 2021 to 2022

The COVID-19 pandemic related decrease in activity in autologous HCT for the majority of disease indications observed in 2020 had mostly resolved in 2021. However, in 2022 a slight decrease of −1.7% in overall autologous HCT activity was reported (+3.9% in 2021). The main indications for autologous HCT were lymphoid malignancies (90%) with PCD comprising 57.1% of all autologous HCT indications (*n* = 13,694). Autologous HCT activities for lymphoproliferative disorders continued to increase by +2.4% for PCD (+4.8% in 2021) and declined for NHL by −10.5% (+4.3% in 2021) (Fig. 3b). Of the autologous transplants for NHL (*n* = 5679), the majority were for DLBCL (all types) (*n* = 2841; 50.0%), other B cell NHL (*n* = 2031; 35.8%) and a minority had T-cell NHL (*n* = 807; 14.2%) as the reported indication. A decrease was observed in both HL by −3.4% (+7.5% in 2021) and ALL by −28.6% (+3.7% in 2021).

In solid tumors, autologous HCT numbers decreased slightly again from 1635 in 2021 to 1563 in 2022 (−2.6%) and have remained stable for the last 10 years. For all types of AML, the decrease in autologous HCT activity has continued in 2022 by −2.8% (−4.5% in 2021). For autoimmune diseases, the overall decrease of −44.7% seen in 2020 recovered in 2021 with an increase of +57.0% (*n* = 468) and is comparable to the reported activity in 2022 (*n* = 464; −0.9%). The main indication for autologous HCT in AID is multiple sclerosis (*n* = 380). Although the numbers overall are still lower than in 2018 (*n* = 550), the decrease seen in 2020 followed by an increase in both 2021 and 2022 was most likely related to the SARS-CoV-2 pandemic according to the EBMT guidelines specifically developed for AID during the pandemic phase [20, 21].

### Transplant activity rates by country

Assessing transplant rates per 10 million population (TR/10^7^) allowed the comparison of activity in countries adjusted for differences in population size. In the 2022 survey, the TR rates for allogeneic HCT within European countries only ranged from 3.1/10^7^ in Bosnia and Herzegovina to 440.8/10^7^ in Israel, followed by 394.7/10^7^ in Germany, 351.2/10^7^ in Belgium, 340/10^7^ in the Netherlands, and 325/10^7^ in Switzerland. The median number of total allogeneic HCT by country was 106 and median TR 138.7/10^7^. Four countries did not report any allogeneic HCT (Cyprus, Georgia, Iceland, and Luxembourg). For autologous HCT, rates ranged from 1.0/10^7^ in Azerbaijan to 598/10^7^ in Slovenia followed by 581.1/10^7^ in Lithuania, 575.1/10^7^ in Italy, 548.6/10^7^ in the Netherlands, and 547.9/10^7^ in Norway. The median number of total autologous HCT by country was 170 and median TR 233.3/10^7^. All countries participating in the annual survey reported doing autologous HCT (see Supplementary Fig. 1a: transplant rates for allogeneic HCT and 1b: transplant rates for autologous HCT).

### Advanced cellular therapy products (ATMPs) and DLI

Table 2 shows the number of patients who received advanced cellular therapy and DLI in 2022. Unmanipulated DLI infusions were reported in 2854 patients, a decrease of −12.1% compared to 2021. The majority of DLI were given for relapse (*n* = 1294) and graft enhancement/failure (*n* = 804).

**Fig. 4:**
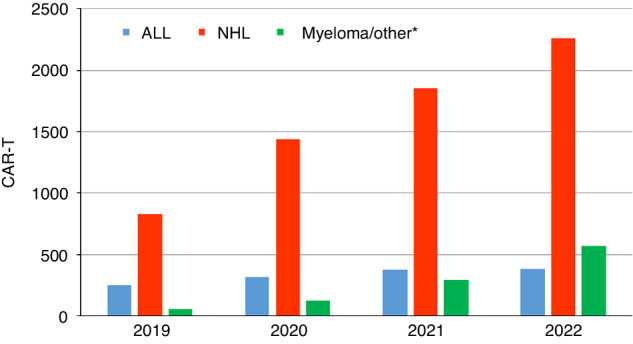
Absolute number of patients receiving CAR-T cellular therapy by indication from 2019 to 2022.

A total of 4329 patients (+23.9%) in 289 centers from 32 countries received forms of hematopoietic cellular therapies that qualified as medicinal products rather than cell transplants (https://www.ema.europa.eu/en/documents/scientific-guideline/qualification-opinion-cellular-therapy-module-european-society-blood-marrow-transplantation-ebmt_en.pdf). In 2022, the ongoing and impressive increase is again observed for gene-modified T-cells, notably CAR-T cells, increasing from 301 reported in 2018 to 1134 in 2019, 1875 in 2020, 2524 in 2021 and 3205 in 2022, an overall 10-fold increase (Fig. 4). The main increase seen by disease is for myeloma/others, increasing from 56 in 2019 to 566 in 2022. This is followed by NHL, increasing from 826 in 2019 to 2259 in 2022 and ALL, increasing from 252 in 2019 to 380 in 2022. The numbers of patients treated with CAR-T cells has increased constantly since 2018 and does not seem to be impacted by the SARS-CoV-2 pandemic. Two hundred and fourteen centers in 28 countries reported 3205 CAR-T cellular therapies in 2022. Almost all centers that offer CAR-T cell therapy reported experience in both allogeneic and autologous HCT. Of the 214 CAR-T cellular therapy centers, just 15 reported doing autologous HCT only and 4 centers allogeneic HCT only. We cannot exclude that there are centers that perform CAR-T cellular therapies but not allogeneic or autologous HCT that do not report to the EBMT, but we assume that the number is low. The main indication for CAR T therapy in 2022 was lymphoma (*n* = 2259; 70.5%), followed by myeloma (*n* = 470; 14.7%), ALL (*n* = 380; 11.8%), and other malignancies (*n* = 96; 3.0%) [22–30]. Use of CAR-T technology continues to increase. High rates are reported from countries with a high gross national product including Israel 231 per 10 million inhabitants followed by Switzerland 145/10^7^, France 89/10^7^, Netherlands 80/10^7^, and Germany 79/10^7^ (see supplementary figure 1c). The median number of patients receiving CAR-T therapy reported by country was 28 (range 1–221) and median CTR 33.1/10^7.^ Fifty-two patients receiving an allogeneic CAR-T therapy were reported by 17 centers in twelve countries. For autologous CAR-T, 3153 (98.4%) patients were reported by 214 centers in 28 countries. The number of reported CAR-T therapies performed in Europe is increasing steadily and has reached a cumulative total of 9039 patients treated between 2018 and 2022 [31]. Finally, we attempted to analyze whether the increase in CAR-T cell activity was accommodated by allocating more resources to specific centers or by cutting established activities. For this purpose, we analyzed the 25 centers reporting 30 or more CAR-T therapies in 2022. These centers were from 8 countries. Twenty -three centers performed both allogeneic and autologous HCT in addition to CAR-T treatment, while 2 centers performed autologous HCT only. The results are heterogenous i.e., while in some centers autologous and allogeneic transplant activity decreased as CAR-T activity increased this was not observed in other centers.

The second most widely used cellular therapy in 2022 other than CAR-T was mesenchymal stromal cell therapy (*n* = 355; 93% allogeneic), their use being mainly to treat graft-versus-host disease [32]. Promising results with mesenchymal stromal cell therapy have also been reported in autoimmune diseases [33]. Numbers of other cellular therapy products have not greatly changed since 2019. Of note, specific data on tumor infiltrating lymphocytes is not collected separately in the annual survey.

## Discussion

The EBMT activity survey has been conducted annually since 1990 [1]. Over 46,000 transplants in almost 42,000 patients were reported in 2022. The largest number of transplants ever reported was in 2019 (48,412 in 43,581 patients). In 2020, a considerable decrease in transplant activity was observed, most likely due to the SARS-CoV-2 pandemic. In 2021 transplant activities improved, despite the ongoing pandemic. To our surprise, the number of transplants performed in 2022 decreased again. The reason for this observed decline is unclear. Reasons may include, availability of alternative therapies, competition within centers between transplant and CAR-T treatment, staff shortage and resource limitations after the pandemic ended or potential continued effects related to the pandemic. The observation that transplant activities declined across all indications and across all donor types (autologous and allogeneic) suggests a general rather than a disease specific cause. The upcoming annual surveys of transplant activities will reveal whether the continued increase in transplant activity observed from 1990 to 2019 has slowed down definitively or only temporarily.

In allogeneic HCT, donor choice demonstrated a continued trend that is moving away from HLA-identical family donors and possibly haplo-identical donors, while the use of unrelated donors seems to have stabilized. The use of cord blood donors is at a very low level.

The use of DLI, which had increased continuously over the last 20 years and almost tripled over this time period has shown a drop of −12% in 2022. The reasons for this are not well understood.

Some countries had difficulties in reporting data, possibly related to either decreasing activity or the incapacity to report due to personnel shortage. This amounts to approximately 1500 patients who in previous years were reported as having received an allogeneic or autologous HCT. Adding the numbers of potentially missing transplants should not change the overall trend of decreasing activity in allogeneic (−4%) or autologous (−1.7%) HCT in 2022. This contrasts dramatically with the increase in the use of CAR-T cell therapy, that was most pronounced for NHL and MM.

The decrease in allogeneic and autologous HCT activity in lymphoid malignancies may be attributed to the new therapeutic options that have become available, including small molecules, monoclonal antibodies, bispecific antibodies and most notably, CAR-T cells. The increasing popularity of CAR-T cell therapy does however not account for all decline in autologous and allogeneic transplant activity for lymphoid malignancies. The number of autologous HCT for multiple myeloma continues to increase, despite the increased use of CAR T cell therapy for this indication. It could possibly be explained by use of autologous HCT as a first-line treatment in myeloma less affected by the new developments of CAR-T and bispecific antibodies used in more advanced disease stages. In contrast, autologous transplant activity for NHL is slightly decreasing. Overall, the use of CAR-T cell therapy continued to increase in 2022. High rates were reported from countries with a high gross national product.

Heterogeneity in the number of CAR-T treatment per center in comparison to decreasing or stable transplant numbers in the same center may be interpreted as diverting resources to CAR-T activity in some centers in others additional resources must have been made available.

The development of CAR-T technology has important pharmacoeconomic implications with an estimated minimum cost per treatment of €250’000 European wide costs are estimated at €2.25 billion. Costs of CAR-T treatment is not limited to product costs, but patient costs include next to the product the pre-CAR-T phase, the inpatient treatment and post CAR-T treatment costs which have been estimated in a recent paper by Swiss health insurers to amount to €215’000 excluding the product costs. A considerable part of this cost is occurring in the post-CAR-T phase [34]. Given this data total costs for a CAR-T treatment are estimated to be higher than just the product costs and await detailed analysis. Unfortunately, we are lacking data on the percentage of commercially produced as opposed to noncommercial products, but we assume that the contribution of noncommercial products is minor [35].

In summary, we continue to see an impressive increase in the use of CAR-T cell therapy, while growth in allogeneic and autologous HCT has slowed down. Mainly allogeneic and to a lesser degree autologous HCT for lymphoid neoplasia, lymphomas and myelomas are used less, possibly due to the introduction of other alternative therapies. We have previously shown that newer technologies become available in resource rich countries and these rapid developments are expected to widen the gap between patients in resource rich versus resource poorer countries.

The annual activity survey of the EBMT reflects current activity and trends in the field of transplant technology. Despite EBMT recommendations for indications for transplant aiming to standardize practice [4], there appears to be no ‘ideal’ transplant activity rate across countries, even with similar economic strength are considered. Ongoing studies using the EBMT benchmarking model with registry and survey data aim to assess the impact of international variation in activity and clinical practice across countries with similar and variable economies on survival outcomes. This report is valuable for the dissemination of the most recent information on indications, donor, and stem cell usage, which will ultimately be beneficial in health care planning.

### Supplementary information

Supplementary figure 1a

Supplementary figure 1b

Supplementary figure 1c

Appendix of participating centers

## Data Availability

Datasets may be available upon request via EBMT Partnering (partnering@ebmt.org).
