# Supplementary figures and images for: Hematopoietic cell transplantation and cellular therapies in Europe 2022. CAR-T activity continues to grow; transplant activity has slowed: a report from the EBMT
Source: Bone Marrow Transplant. 2024 Mar 4;59(6):803–12. doi: 10.1038/s41409-024-02248-9 (PMC11161408; doi:10.1038/s41409-024-02248-9)

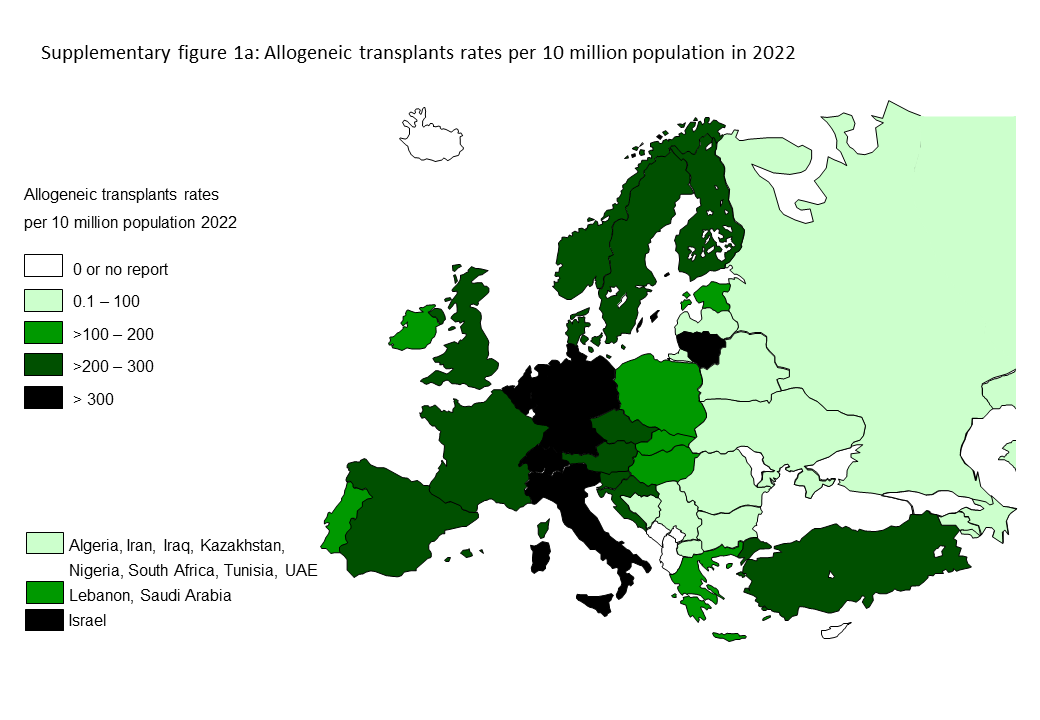

Supplement: Supplementary file 1 — Supplementary figure 1a [file 41409_2024_2248_MOESM1_ESM.tif]

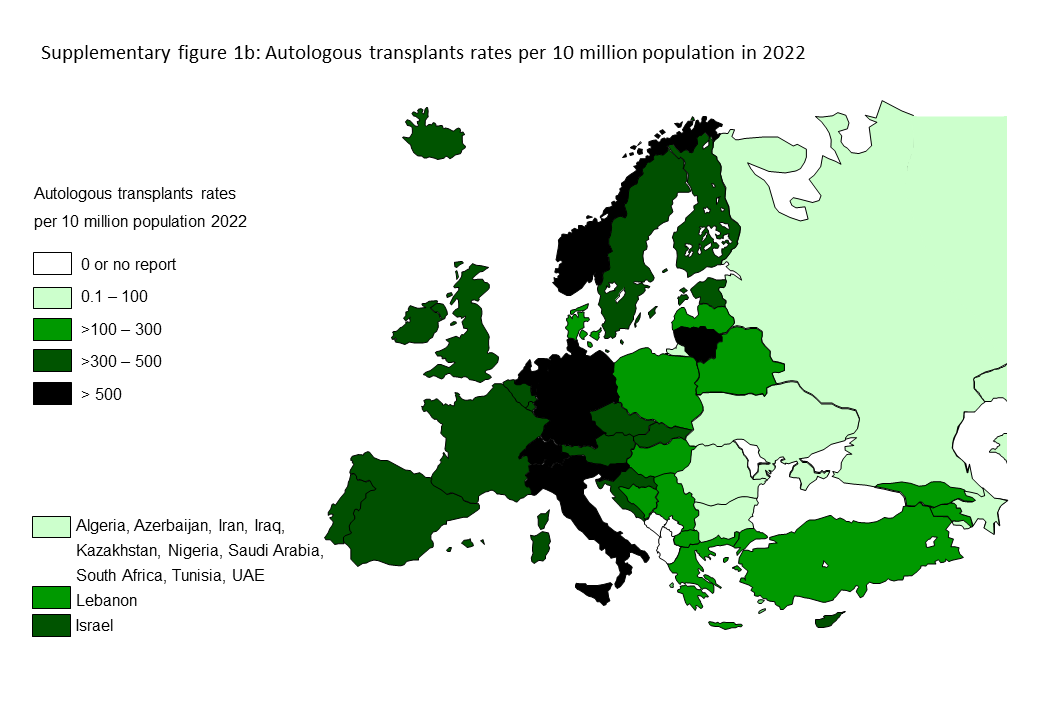

Supplement: Supplementary file 2 — Supplementary figure 1b [file 41409_2024_2248_MOESM2_ESM.tif]

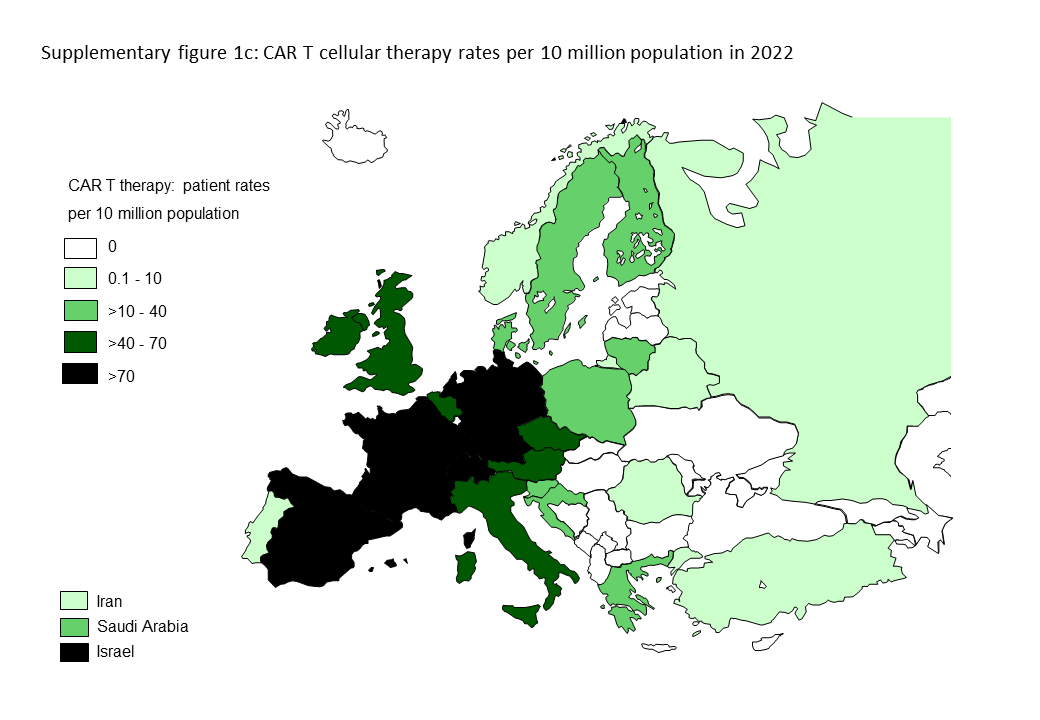

Supplement: Supplementary file 3 — Supplementary figure 1c [file 41409_2024_2248_MOESM3_ESM.tif]
